# Supplementary material for: Citrus Honey Ameliorates Liver Disease and Restores Gut Microbiota in Alcohol–Feeding Mice
Source: Nutrients. 2023 Feb 21;15(5):1078. doi: 10.3390/nu15051078 (PMC10005585; doi:10.3390/nu15051078)

Supplementary Materials:

Table S1. Linear gradient elution program.

Time(min)	0	2	5	10	12	14	15	17	17.5	21
**Mobile phase A (%)**	95	90	60	30	20	20	0	0	95	95
**Mobile phase B (%)**	5	10	40	70	80	80	100	100	5	5

Table S2. Retention time, mass-to-charge ratio, standard curve, and R^2 of SCFAs standards.

SCFAs	RT（min）	mass-to-charge ratio (*m*/*z*)	Regression equation	R^2
Acetic acid	5.27	117	y= 3.7000x + 838004.87	1.0000
Propionic acid	7.19	131	y = 1.3721x + 17843	0.9976
Butyric acid	8.94	145	y = 1.6716x + 15802	0.9991
Valeric acid	11.07	159	y = 1.6041x + 4472.6	0.9956

Table S3. Contents of 26 compounds in CH (μg/100 g honey)

Compounds	Rt min	Concentration （μg/100 g）					Regression equation	R^2
		M1	M2	M3	M4	M5		
4-Hydroxyquinoline	2.79	nd	9.84	7.30	8.16	11.25	y = 7044.30x + 10426.00	0.9975
Salicylic acid	3.26	15.59	14.27	11.92	12.99	13.99	y = 148.13x - 527.70	0.9987
Vanillic acid	3.66	2.79	2.19	1.89	nd	2.10	y = 31.90x + 77.56	0.9999
Rutin	4.09	81.66	73.32	72.78	62.88	75.39	y = 2,074.22 x - 4,781.76	0.9999
Trans-4-hydroxycinnamic acid	4.28	nd	9.84	7.30	8.16	11.25	y = 27.28x + 155.28	0.9999
Ellagic acid	4.38	1.65	1.08	0.86	nd	1.09	y = 33.06x + 114.51	0.9998
Sinapinic acid	4.58	5.79	5.55	nd	2.47	1.80	y = 35.75x + 787.18	0.9948
Luteolin	4.60	5.76	8.41	7.08	5.98	9.29	y = 21.97x + 285.40	0.9990
Trans-ferulic acid	4.61	0.61	0.58	0.54	0.56	0.58	y = 456.32x + 6585.80	0.9970
Hesperidin	4.69	11.22	11.26	9.37	10.35	10.96	y = 167.87x + 2436.60	0.9998
Naringin	4.69	0.37	0.35	0.34	0.35	0.36	y = 1726.70x + 26406.00	0.9916
4-hydroxybenzoic acid	4.83	0.59	0.65	0.41	0.50	0.56	y = 429.43x + 5119.90	0.9958
3,4-Dimethoxycinnamic acid	5.27	111.27	106.48	89.60	94.54	106.49	y = 76.91x + 1534.80	0.9962
3,4-Dihydroxybenzoic acid	5.27	2.58	2.23	2.82	2.79	1.95	y = 181.24x - 3752.40	0.9949
Methyl syringate	5.33	4.53	4.17	3.13	4.08	4.68	y = 218.77x + 695.69	0.9991
Fisetin	5.48	0.48	nd	0.39	0.39	0.37	y = 2141.10x - 4015.30	0.9990
Abscisic acid	5.50	276.20	273.72	235.56	253.07	265.25	y = 470.01x + 5429.20	0.9907
Quercetin	5.58	1.78	1.72	1.66	1.78	1.45	y = 766.94x - 3741.7	0.9998
Trans-cinnamic acid	5.67	1.30	4.20	1.10	2.25	1.23	y = 23.04x + 312.50	0.9962
Naringenin	5.95	0.03	0.03	0.02	0.03	0.03	y = 1954.90x + 357.64	0.9997
Apigenin	6.03	0.58	nd	nd	2.47	2.47	y = 2121.90x - 5531.50	0.9993
Kaempferol	6.15	3.00	2.87	2.96	3.02	2.90	y = 1896.40x - 12028.00	0.9993
Hesperetin	6.17	3.10	2.79	1.78	2.34	3.02	y = 2328.60x - 1106.10	0.9999
Kaempferide	6.29	nd	nd	nd	nd	8.73	y = 15.84x - 4.91	0.9992
Daidzein	7.42	0.12	0.11	0.11	0.11	0.12	y = 3512.10x - 8727.90	0.9992

Compounds	Rt min	Concentration （μg/100 g）					Regression equation	R^2
		M1	M2	M3	M4	M5		
Chrysin	7.70	7.51	7.56	7.54	7.56	7.51	y = 993.78x - 24138.00	0.9966

nd means not detected

Table S4 Histopathological Assessment Grading

Parameter	PF	AF	PC	LH	MH	HH	FG
Steatosis	0	4	1	1	2	2	4
Inflammation	0	2	1	1	2	1	2
Hepatic fibrosis	0	0	0	0	0	0	1
Total	0	6	2	2	4	3	7

	Hepatic disease	Scores
**Steatosis**	Nonsteatosis	0
	Range of steatosis<30%	1 +
	Range of steatosis>30%	2 +
	Range of steatosis>50%	3 +
	Range of steatosis>75%	4 +
Inflammation	No inflammation present	0
	Ballooning degeneration of liver cells<30%	1 +
	Ballooning degeneration of liver cells>30%	2 +
	Ballooning degeneration of liver cells>50%	3 +
	Ballooning degeneration of liver cells>75%	4 +
Hepatic fibrosis	Non-fibrosis	0
	Hepatic fibrosis<30%	1 +
	Hepatic fibrosis>30%	2 +
	Hepatic fibrosis>50%	3 +
	Hepatic fibrosis>75%	4 +

Table S5. The composition table of dominant species in each group of mouse intestinal flora phylum levels.

Phylum	PF	AF	PC	LH	MH	HH	FG
Bacteroidota	0.69%	48.39%	64.56%	64.52%	53.89%	57.71%	48.60%
Firmicutes	23.50%	39.96%	26.24%	25.64%	36.27%	32.13%	35.85%
Campilobacterota	2.06%	1.97%	3.00%	3.65%	3.94%	3.64%	8.60%
Actinobacteriota	2.18%	4.19%	2.49%	0.44%	2.42%	2.62%	1.52%
Verrucomicrobiota	1.52%	2.90%	2.46%	1.16%	0.18%	1.79%	3.36%
Proteobacteria	0.33%	1.31%	0.30%	3.91%	2.70%	1.61%	0.63%
Desulfobacterota	0.71%	0.80%	0.74%	0.29%	0.31%	0.32%	1.12%
Firmicutes/Bacteroidetes ratio (F/B)	33.88%	82.57%	40.64%	39.74%	67.30%	55.67%	73.77%

Figure S1. The base peak intensity chromatogram (BPI) of CH by UPLC-Q-TOF-MS

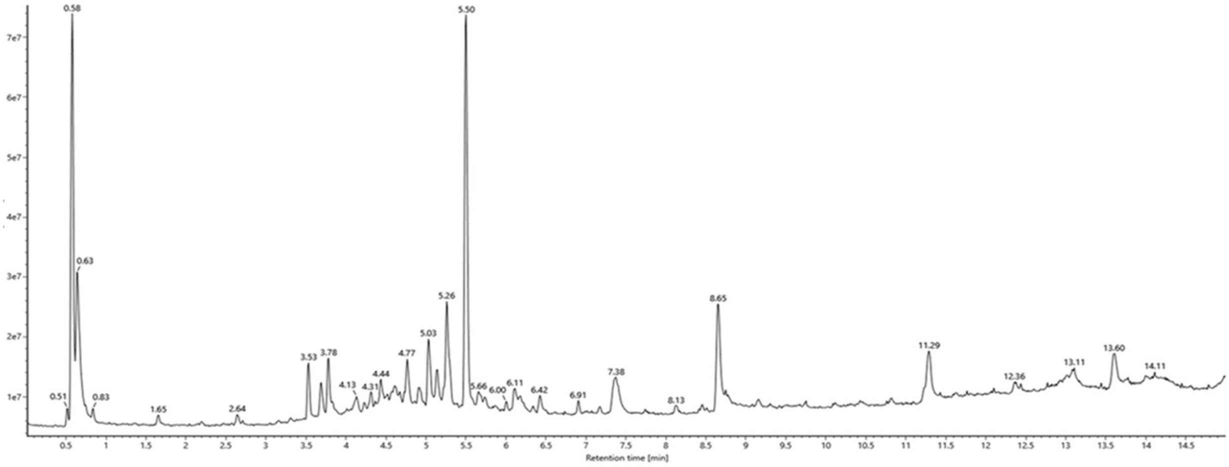

Figure S2. (A) Rarefaction analysis and (B) Shannon index of gut microbiota from seven groups of mice.

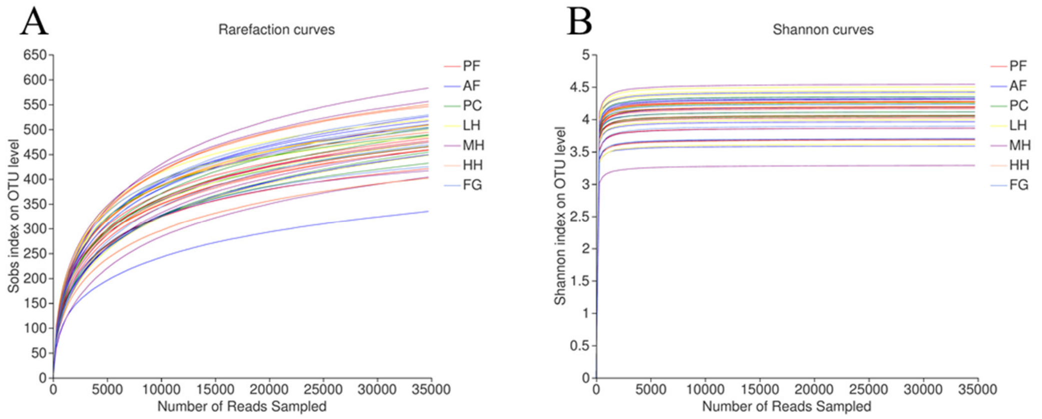

Figure S3. Comparison of the differences of gut microbiota diversity index among seven groups of mice. Chao index (a), Ace index (b), Shannon index (c), and Simpson index(d).

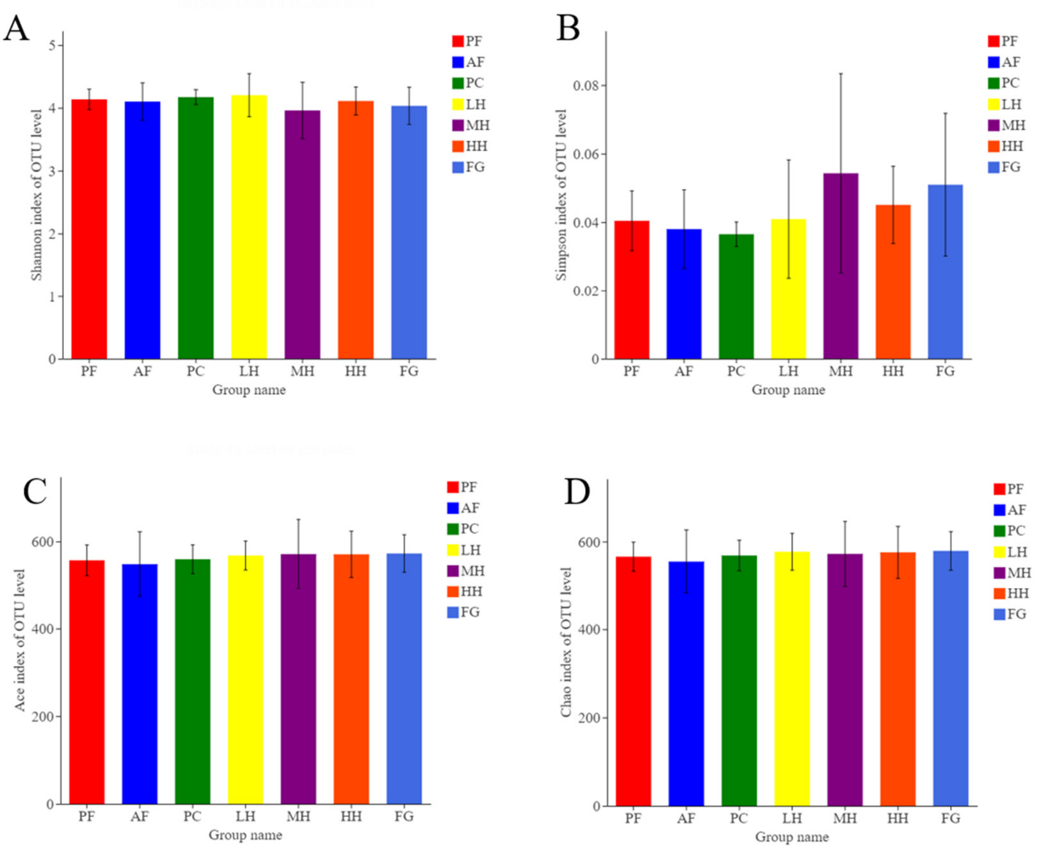

Supplement: Supplementary file 1 [file nutrients-15-01078-s001.zip › nutrients-2124572-supplementary.pdf]
